# Effect of Early Psychological Counseling for the Prevention of Posttraumatic Stress Induced by Acute Coronary Syndrome at Long-Term Follow-Up

**DOI:** 10.3389/fpsyt.2022.846397

**Published:** 2022-05-30

**Authors:** Mary Princip, Aju P. Pazhenkottil, Jürgen Barth, Ulrich Schnyder, Hansjörg Znoj, Jean-Paul Schmid, Rebecca E. Langraf-Meister, Roland von Känel, Katharina Ledermann

**Affiliations:** ^1^Department of Consultation-Liaison Psychiatry and Psychosomatic Medicine, University Hospital Zurich, University of Zurich, Zurich, Switzerland; ^2^Department of Cardiology, University Hospital Zurich, University of Zurich, Zurich, Switzerland; ^3^Cardiac Imaging, Department of Nuclear Medicine, University Hospital Zurich, University of Zurich, Zurich, Switzerland; ^4^Institute for Complementary and Integrative Medicine, University Hospital Zurich, University of Zurich, Zurich, Switzerland; ^5^Department of Clinical Psychology and Psychotherapy, University of Bern, Bern, Switzerland; ^6^Department of Cardiology, Clinic Gais, Gais, Switzerland; ^7^Clienia Schlössli AG, Oetwil am See, Switzerland; ^8^Department of Clinical and Health Psychology, University of Fribourg, Fribourg, Switzerland

**Keywords:** posttraumatic stress symptoms, myocardial infarction, early prevention, counseling, acute trauma stress, acute coronary care, behavioral cardiology

## Abstract

**Objective:**

Psychological consequences of myocardial infarction (MI) are substantial, as 4% of all MI patients develop posttraumatic stress disorder (PTSD) and 12% clinically relevant posttraumatic stress symptoms (PTSS). The study investigated the course and development within 12 months of MI-induced PTSS to gain novel insights in potentially delayed response to early trauma-focused counseling aimed at preventing the incidence of MI-induced PTSS.

**Methods:**

In the MI-SPRINT two-group randomized controlled trial, 190 MI-patients were randomly allocated to receive a single-session intervention of either trauma-focused counseling or an active control intervention targeting the general role of stress in patients with heart disease. Blind interviewer-rated PTSS (primary outcome) and additional health outcomes were assessed at 12-month follow-up.

**Results:**

12-month follow-up of outcomes were available for 106 (55.8%) of 190 participants: In the entire sample, one patient (0·5%, 1/190) who received trauma-focused counseling developed full PTSD. There was no significant difference between trauma-focused counseling and stress counseling regarding total score of interviewer-rated PTSS (*p* > 0.05). The only group difference emerged in terms of more severe hyperarousal symptoms in the trauma-focused counseling group in the ITT analysis, but not in the completer analysis.

**Conclusions:**

No benefits were found for trauma-focused counseling after 12 months when compared with an active control intervention. PTSD prevalence in the present study was low highlighting a potential beneficial effect of both interventions. Further studies are needed to determine the most accurate approach of counseling.

## Introduction

Posttraumatic stress disorder (PTSD) is a mental disorder that may occur in the aftermath of a traumatic or stressful, life-threatening event, involving high intensity of fear of dying, helplessness and loss of control (1). About 70% of patients experience psychological responses including moderate to intense fear of dying and distress during an acute myocardial infarction (MI) (2). The psychological consequences of MI are substantial, as 4% of all MI-patients develop full PTSD, and clinically relevant posttraumatic stress symptoms (PTSS) are found in 12% (3). Re-experiencing the acute cardiac event, avoidance of cardiac-related stimuli, persistence of negative feelings and thoughts, as well as hyperarousal and reactivity, are typical features of MI-induced PTSS. In order to meet diagnostic criteria for PTSD, symptoms have to be present for at least 1 month and must significantly interfere with a patient's daily functioning (1).

There is an intriguing line of recent research suggesting that clinically relevant PTSS are predictive of incident cardiovascular disease (CVD) morbidity and mortality and may also worsen cardiovascular prognosis (4–8). Among poor quality of life and impaired mental health, MI-induced PTSS is associated with a twofold increased risk of adverse clinical outcomes, including major adverse cardiac events, cardiac hospital readmissions, and all-cause mortality (8, 9). Poor cardiac prognosis can be attributed to either unfavorable health behaviors such as physical inactivity, concomitant sleep problems, poor adherence with cardiac medication and non-attendance in cardiac rehabilitation programs, as well as through direct pathophysiological and cardiometabolic effects, including endothelial dysfunction, dyslipidaemia, inflammation and hypercoagulability (9–12).

Early interventions to lower the incidence of MI-induced PTSS are of high relevance but are scarce due to a lack of appropriate interventions for these patients (9). Interventions such as debriefing at an early stage aim to reduce stress reactions within several hours or days of trauma exposure. However, debriefing does not prevent the occurrence of PTSD (13, 14) and can even be harmful (15) when focusing on the emotional re-experiencing of the trauma itself (16).

Alternatively, early interventions after trauma for patients with MI might aim at reducing helplessness, and fear of dying (14). To achieve alleviation of these symptoms, reassurance of the MI patient that he or she shows a normal psychological reaction to an abnormal situation, providing a secure place, reducing the exposure to further stress, taking care of physiological needs, providing information and orientation about possible (posttraumatic) stress reactions, recruiting social support, and emphasizing the expectation of returning to normality should be provided (14).

A recent systematic review and meta-analysis on randomized controlled trials (RCT) of early psychological interventions designed to prevent PTSS found a benefit only if treatment was provided to trauma survivors who were identified as having a high risk of developing clinically significant PTSS based on early predictors of PTSD (17). Perceived threat, fear of dying and helplessness during MI, as well as depressive and acute stress symptoms, were shown to increase the risk for interviewer rated PTSS 1 month after the event in about 400 MI-patients (18). Moreover, fear of dying and sense of helplessness predicted MI-induced PTSS 3 months after the cardiac event in 394 patients (19). Therefore, a high level of distress during MI, characterized by fear of dying, helplessness and acute pain can indicate whether patients are at high risk to develop MI-induced PTSS (18–21).

Based on these previous findings, the primary focus of our RCT “Myocardial Infarction-Stress PRevention INTervention (MI-SPRINT)” was to investigate whether the development of PTSS can be reduced by a single counseling session that is administered early on (i.e., within 48 h) after MI to patients at high risk to develop PTSD (those with relevant chest pain, fear of dying, and helplessness, all during MI) within a setting of a Coronary Care Unit (CCU) (22).

Our primary hypothesis was that posttraumatic stress levels at the 3-month follow-up would be at least 20% lower in the intervention group than in the control group (22, 23). Results from the 3-month follow-up intention-to-treat analyses showed no difference in interviewer-rated PTSS between trauma-focused counseling (mean, 11.33; 95% Cl, 9.23–13.43) and stress counseling (9.88; 7.36–12.40; *p* = 0.40). Therefore, the aim of the present study was to investigate the development and course of PTSS within 12 months after MI to gain novel insights in potential delayed responses to the intervention beyond 3 months. Additionally, mental and physical health outcomes were analyzed for secondary group comparisons.

## Materials and Methods

### Study Participants and Trial Design

This is a secondary analysis of data from 106 participants in the MI-SPRINT RCT who completed the 12-month assessments of interviewer-rated PTSS. The study protocol and the 3-month follow-up of this RCT have previously been published (22, 23). The study protocol was approved by the ethics committee of the State of Bern (KEK No. 170/12) and registered under ClinicalTrials.gov (NCT01781247). All patients gave written consent to study participation. None received any financial compensation.

We invited consecutive patients with verified acute ST-elevation myocardial infarction (STEMI) or non-STEMI, referred for acute coronary care intervention to the Cardiology Department, Bern University Hospital, Switzerland, between February 2, 2013 and September 29, 2015, to participate in the MI-SPRINT trial. Inclusion criteria were age 18 years or older; stable circulatory conditions, and high level of acute distress during MI. Acute distress during MI was assessed using numeric rating scales (scores 0–10) for “pain intensity (during MI),” “fear of dying” and “making sorrows and feelings helpless” (e.g., Please rate from 0 to 10: “When the study therapist told me I was having a heart attack, I did worried and felt helpless).

High acute distress during MI was defined a priori on the basis of a score of at least 5 for pain plus at least 5 for fear of dying and/ or helplessness. Exclusion criteria included emergency coronary artery bypass grafting; comorbid disease likely to cause death within 1 year; not fully oriented to situation, person, and place; cognitive impairment per an adapted short version of the Mini-Mental State Examination (score <7; maximum score = 9); current severe clinical depression according to medical history, suicidal ideations in the previous 2 weeks, inadequate knowledge of German; or current participation in another RCT.

### Randomization and Masking

This RCT was a single-blinded 2 parallel group behavioral RCT with 2 active face-to-face interventions of the same duration and attention. The study was performed by the bedside within 48 h after patients had reached stable circulatory conditions within the premises of the coronary care unit. Patients were randomly allocated to trauma-focused counseling (intervention group) or stress counseling (control group). Randomization was performed with an online tool (www.Randomizer.org) that created a list for the group allocation. The intervention assignment was single-blinded such that the list was available to investigators only after the 12-month follow-up assessment of the last patient.

### Interventions

The interventions were delivered by five counselors with either a bachelor's degree in psychology or medicine. Each of the five persons delivered both interventions.

Supervision was performed by senior clinical psychotherapists with master/doctor degrees in psychology or psychiatry. Each intervention consisted of one single session of individual counseling for a duration of 45 min, based on colored information booklets, which were tailored for both interventions (online suppl. Booklets; see www.karger.com/doi/10.1159/000486099 for all online suppl. Material). The booklets were used for interacting with the patient during the counseling session and to deepen specific topics. To get examples of specific topics that could be covered during the 45 min counseling in each group see (23). The structure of the counseling session was for both groups the same. In brief, there was a 5-min introduction phase, during which the patient was informed about the setting of the study, which was followed by either 40-min trauma-focused counseling or stress counseling. Sessions could be paused for medical reasons and later resumed on short notice. In the trauma-focused counseling group, the concept of psychological trauma and symptoms of PTSD were explained using an educational and resource-oriented approach. In the stress counseling group, patients received information about the general role and consequences of psychosocial stress and stress management techniques in order to promote health behavior and everyday functioning after MI, but any trauma-related terminology was strictly avoided. The trauma-focused intervention was thoroughly described in the original paper (22). In short, the first step in the trauma-focused intervention group was to normalize any emotional reaction following the heart attack and explain the concept of psychological trauma and symptoms of PTSD. Questions like e.g., “Why can a heart attack be traumatizing? What is a trauma? What are typical (i.e., trauma-related) symptoms that may occur?” were addressed. In the second part of the counseling session, the study therapists offered practical coping strategies that could be applied should symptoms of PTSD occur after MI. Strategies to deal with posttraumatic symptoms such as avoidance, safety behaviors, anxiety, anger/irritability and sleeping problems were addressed. To activate resources of the patients, they were asked e.g., “Do you know any strategies that have helped you when you had this problem (e.g., stressful situations, sleeping problems, anxiety) in the past? Do you have places where you feel safe?” Afterwards, helping strategies, e.g., to establish a safe place, breathing techniques to reduce hyperarousal and anxiety symptoms were accomplished.

### Baseline Measures

All study patients underwent a structured medical history and psychometric assessment before the intervention. Additional information was gathered from patients' files. Education was categorized as university graduation, including applied sciences/high school graduation; apprenticeship or vocational school; lower than apprenticeship or vocational school. Body mass index was calculated based on the weight and height disclosed by patients. Smoking was assessed in terms of current, former or never smokers. Information on diabetes, hypertension, high cholesterol and previous MI were obtained from patient files. Left ventricular ejection fraction was taken from angiography records. The Global Registry of Acute Coronary Events (GRACE) risk score was used to determine the risk of post-discharge death and recurrent MI after ACS (24). Depression was assessed in terms of life-time depression history taken from the patients records and the 13-item cognitive depressive symptom subscale of the Beck Depression Inventory (BDI; total score 0–39) (25), a reliable assessment tool for depressive symptom levels in patients with coronary heart disease (26). In patients who completed all 13 items, Cronbach's α for the scale was 0.66, indicating moderate internal consistency. Use of antidepressant medication was obtained from the patients' record. Acute distress during acute MI was assessed using a three item (individual scores of pain intensity, fear of dying and helplessness during MI) numeric rating scale (range 0–10) that summed the three items and divided the total by three to obtain a severity score (range 3.33–10) (19). The German version of the 19-item self-rating Acute Stress Disorder Scale was used to self-rate symptoms of dissociation, re-experiencing, avoidance, and arousal that had occurred since MI. Items were rated on a 5-point Likert scale (ranging from 0 =”not at all”, to 4 =”extremely” yielding a total score from 0 to 76) (27). In patients who completed all 19 items, Cronbach α for the scale was 0.82, indicating good internal consistency. PTSD cases as a consequence of traumatic experiences in the 3 months prior to current MI were explored with a 3-item screener (28).

### Outcome Measures

All patients were invited to participate in the outcome assessment 12 months after MI. The assessors of the outcome measures were blinded to group assignments. The primary outcome of the MI-SPRINT trial was the total score of the validated German version of the Clinician-Administered PTSD scale (CAPS) (29, 30) with reference to Diagnostic and Statistical Manual for Mental Disorders DSM-IV criteria (31), in official use at the time we planned the trial. Frequency and intensity of each of the 17 PTSS in the prior month between 0 (never) and 4 (almost always) was assessed by an interviewer resulting in a total PTSS severity score (range 0–136). The CAPS was scored by the same person who also performed the interview. In patients who completed all 19 items, Cronbach's α for the scale was 0.82, indicating good internal consistency. As a secondary outcome, we used the 17-item Posttraumatic Diagnostic Scale (PDS) to rate the severity of self-rated MI-induced PTSS in the last month (total score 0–51) (32, 33). Further secondary outcomes included depressive symptoms (total score of cognitive and somatic symptoms combined 0–63) and global psychological distress (total score 0–36), measured respectively, with the 21-item BDI (25) and the 9-item short form of the Symptom Checklist-90-Revised (SCL-R) (34).

### Power Analysis

We selected the CAPS total score as our primary outcome measure because interviewer-diagnosed PTSS are more clinically meaningful than self-reported symptoms. However, due to the lack of CAPS data in the literature on the incidence of PTSS after MI, reflecting the usual care of this patient population, the power analysis was based on our previously published self-report PDS data (22, 35). We assumed that patients with a high risk of developing PTSD show a 2.9 ± 10.1-point difference in PDS scores 3 months after MI, corresponding to a clinically meaningful 20% lower PDS scores in the trauma-focused vs. the stress counseling group. To yield this difference significant with an alpha error level of *p* = 0.05 and a beta error level of 20%, the sample size was determined as *n* = 194 for each group (22). Despite vigorous efforts, this recruitment goal was not achieved, mainly due to the large number of early patient discharges after changes in the rehabilitation in Switzerland's health care system.

### Statistical Analysis

Data were analyzed using SPSS 25.0 for Windows (SPSS Inc., Chicago, IL) with a two-sided significance level of *p* < 0.05. Patients deceased at follow-up were not imputed and therefore, the sample was reduced to 183 for the ITT analysis and 106 cases for the completer analysis. Education, fear of dying, helplessness, acute stress disorder symptom and cognitive depressive symptom scores at baseline were used as predictors for multiple imputations (*k* = 5) and the pooled analysis for the overall findings. Independent *t*-test and Pearson Chi-Square test were used to compare intervention groups on sociodemographic, clinical and psychological variables. A general linear model with group (intervention vs. control) as fixed factor and controlling for sociodemographic (age, sex, education) and clinical baseline characteristics (GRACE score, PTSD screen, lifetime depression and pain intensity during MI) was applied. We conducted the analysis for the primary outcome (CAPS) and all secondary outcomes (PDS, BDI, SCL-90-R).

## Results

### Study Population

A total of 190 patients were randomized to either trauma-focused counseling (*n* = 97) or stress counseling (*n* = 93). The 3-month follow-up assessment was completed by 154 patients (81·1%). The 12-month follow-up was completed by 106 patients (55.8%). We lost 41 participants due to low funding, bad physical condition, unreachability and 7 patients had died during follow-up. Of all patients completing the 12 months follow-up, 59 (55.7%) were in the trauma-focused counseling group and 47 (44.3%) in the stress counseling group. Table 1 shows the baseline characteristics of the subsample. Only one parameter, i.e., fear of dying during MI was significantly higher in the trauma-focused group than in the stress counseling group (*p* = 0.026).

**Table 1:** Baseline characteristics of the 106 study participants per type of intervention.

	**Trauma-focused counseling group (*n =* 59)**	**Stress counseling group (*n =* 47)**	***P*-value for difference**
Age (years)	60.3 ± 10.8	59.5 ± 11.7	0.627
Male gender (%)	81.4	83.9	0.659
Previous MI (%)	8.4	13.0	0.307
ST-elevation MI (%)	72.2	70.3	0.781
Left ventricular ejection fraction (%)	49.1 ± 12.0	46.1 ± 11.6	0.082
GRACE score	107.1 ± 28.0	106.3 ± 25.4	0.439
Body mass index (kg/m^2^)	27.5 ± 4.8	27.9 ± 4.5	0.486
Diabetes (%)	14.9	14.1	0.883
Hypertension (%)	50.5	52.5	0.822
High cholesterol (%)	50.0	41.3	0.234
Current smoker (%)	43.8	44.6	0.910
Pain intensity (NRS)	8.0 ± 1.6	7.8 ± 1.7	0.320
Fear of dying (NRS)	5.7 ± 2.7	4.8 ± 3.1	0.026
Helplessness (NRS)	5.3 ± 2.7	5.6 ± 2.6	0.506
PTSD screen positive (%)	14.3	7.1	0.123
Lifetime depression (%)	27.4	29.3	0.764
Cognitive depressive symptoms	2.8 ± 2.9	2.7 ± 2.8	0.891
Antidepressant medication (%)	10.3	5.4	0.207
Acute stress disorder symptoms	17.1 ± 10.6	15.4 ± 8.9	0.269

*Continuous data represents mean values with standard deviation. GRACE, Global Registry of Acute Coronary Events; MI, myocardial infarction; NRS, numeric rating scale; PTSD, posttraumatic stress disorder*.

### Effectiveness of the Intervention

Primary and secondary outcomes comparing both groups, i.e., trauma-focused counseling and stress counseling, are presented in Table 2. Overall, symptom scores were low in both groups with absolute values that were mostly higher with trauma-focused counseling than stress counseling.

**Table 2:** Differences in primary and secondary outcomes between types of interventions.

	**Trauma-focused counseling**		**Stress counseling**		***P***
	**Mean**	**95% CI**	**Mean**	**95% CI**	
CAPS total (ITT) (*n =* 97/*n =* 93)	10.18	8.36–12.01	8.91	5.84–11.97	0.653
CAPS total (completer) (*n =* 59/*n =* 47)	10.42	8.27–12.57	8.21	5.54–10.89	0.194
CAPS re-experiencing (ITT) (*n =* 97/*n =* 93)	2.45	1.53–3.36	1.88	0.83–2.93	0.501
CAPS re-experiencing (completer) (*n =* 59/*n =* 47)	2.45	2.08–2.82	1.89	1.47–2.31	0.053
CAPS avoidance (ITT) (*n =* 97/*n =* 93)	3.45	2.34–4.54	2.42	1.17–3.68	0.096
CAPS avoidance (completer) (*n =* 59/*n =* 47)	3.46	2.37–4.55	3.89	1.27–3.55	0.187
CAPS hyperarousal (ITT) (*n =* 97/*n =* 93)	4.82	3.78–5.87	4.32	3.13–5.51	0.031
CAPS hyperarousal (completer) (*n =* 59/*n =* 47)	4.49	3.51–5.48	4.09	2.97–5.20	0.584
PDS total (ITT) (*n =* 97/*n =* 93)	5.68	3.81–7.55	4.77	3.12–6.42	0.433
PDS total (completer) (*n =* 49/*n =* 47)	5.73	3.92–7.55	4.75	2.90–6.60	0.454
PDS re-experiencing (ITT) (*n =* 97/*n =* 93)	1.33	0.83–1.83	1.29	0.71–1.86	0.809
PDS re-experiencing (completer) (*n =* 59/*n =* 47)	1.36	1.14–1.57	1.29	1.04–1.53	0.692
PDS avoidance (ITT) (*n =* 97/*n =* 93)	1.85	1.11–2.59	1.37	0.52–2.21	0.378
PDS avoidance (completer) (*n =* 59/*n =* 47)	1.83	1.52–2.14	1.41	1.05–1.76	0.077
PDS hyperarousal (ITT) (*n =* 97/*n =* 93)	2.57	1.88–3.26	2.01	1.22–2.79	0.428
PDS hyperarousal (completer) (*n =* 59/*n =* 47)	2.51	2.22–2.81	2.08	1.75–2.41	0.054
BDI total (ITT) (*n =* 97/*n =* 93)	5.66	4.40–6.93	4.81	3.36–6.25	0.381
BDI total (completer) (*n =* 59/*n =* 47)	5.45	4.91–5.99	5.11	4.49–5.72	0.411
SCL-9 total (ITT) (*n =* 97/*n =* 93)	4.73	3.65–5.81	4.25	2.67–5.82	0.536
SCL-9 total (completer) (*n =* 59/*n =* 47)	4.48	3.06–5.91	4.14	2.86–5.42	0.727

*BDI, Beck Depression Inventory; CAPS, Clinician-Administered Posttraumatic Stress Disorder (PTSD) Scale; ES, effect size; ITT, intention-to-treat; PDS, Posttraumatic Diagnostic Scale; SCL, Symptom Checklist*.

*All analyses were controlled for age, sex, education, Global Registry of Acute Coronary Events score, PTSD screen, lifetime depression and pain during acute coronary syndrome*.

### Primary Outcome

In the entire sample, there was one patient (0·5%, 1/183) in the trauma-focused counseling group who had developed full PTSD 12 months after MI. However, there was no significant difference between trauma-focused counseling and stress counseling regarding the total score of interviewer-rated PTSS, both in the completer and ITT analysis (*p* > 0.05). The groups only differed in the CAPS hyperarousal score in the ITT analysis, with participants in the trauma-focused counseling group showing more severe hyperarousal symptoms than controls. In all other scores of individual PTSS clusters, the groups did not differ significantly, neither in the ITT analysis nor the completer analysis.

### Secondary Outcomes

There were no significant differences in self-rated PTSS between trauma-focused vs. stress counseling. Similarly, there was no significant difference in global psychological distress and depressive symptoms in the trauma-focused vs. stress counseling group neither in the completer nor the ITT analysis.

## Discussion

We previously reported no significant difference in clinician-rated PTSS at 3 months follow-up when compared to an active control intervention in patients at risk to develop MI-induced PTSS (23). The current study extends the follow-up period to 12 months and confirms previous results, i.e., there is no benefit for the prevention of clinician-rated PTSS after MI between patients who had received trauma-focused vs. stress counseling at hospital admission. Moreover, as this finding was confirmed in the ITT analysis and the completer analysis, the results seem to hold true not only for those patients who completed the follow-up assessment at 12 months but also for all patients. Hence, our primary hypothesis that posttraumatic stress levels at the 3-month follow-up will be at least 20% lower in the intervention group than in the control group, and that this effect will last up to 12 months after the intervention was not confirmed by the study results. Moreover, the findings were also negative regarding secondary outcomes, i.e., depressive symptoms and global psychological distress.

In contrast to our previous finding of significantly higher self-rated PTSS with trauma-focused counseling than stress counseling both in the ITT and the completer analysis after three months (23), the current analysis showed no such differences; i.e., self-rated PTSS were not statistically different between groups after 12 months. We offer the following explanation for this discrepant finding. It is possible that traumatic memories of the cardiac event might have been further stimulated by trauma-focused counseling and have framed patients' attribution of stress symptoms as traumatic at 3 months (36), while this effect might have diminished in the course of 12 months. Another explanation of the results presented here and in the previous study (23) might be that one single trauma session of trauma-focused counseling might not yield enough effect for a sufficient intervention to favorably influence the development of self-rated PTSS in the average MI patient at 3 months, while it may have positively altered the long-term course over 12 months.

The principle behind this idea was to provide a pragmatic approach for the busy cardiology setting by designing an intervention that could be easily implemented in daily clinical routine without affecting urgently needed cardiac examination and therapy, such as emergency coronary angiography. It might well be that by choosing several counseling sessions the effect might have been even better. To this end, it is worth mentioning that in a trial on patients who received an implantable cardioverter defibrillator for primary prevention of sudden cardiac death, cognitive behavioral therapy with eight telephone counseling sessions resulted in improvement of self-rated PTSS compared to usual cardiac care after 12 months (37). From a neuro-scientific perspective, different brain areas are activated during the process of a traumatic event. While PTSD patients process traumatic memories more likely in the limbic system, non-PTSD patients are shown to process them in the prefrontal cortex (38). Our intervention was not specifically aimed at altering the traumatic memory process. Early treatments aimed to shift the memory process from limbic to prefrontal cortex were revealed to be successful in preventing PTSS (39). Therefore, further studies should integrate neuro-scientific approaches to reduce PTSS following MI.

When comparing the present study to our previous study (23), the severity of total interviewer-rated PTSS between the 3- and 12-month follow-up assessments was in the same range confirming the findings of earlier studies suggesting that MI-induced PTSS may prevail in the first year after MI and decrease in only some of the patients thereafter (8, 35, 40, 41). However, in most of these previous studies, PTSS was—in contrast to the present study—not assessed by a clinical interview and analyses in terms of individual clusters of PTSS were not performed.

The overall incidence of PTSS in the current study was low compared to previous studies: Only one patient of the entire sample (0.5%) developed PTSD while meta-analytic data show that 4% (95% CI, 3–5%) of patients will develop PTSD after MI (3). One possible reason to explain this might be that both active interventions, i.e., trauma-focused counseling and stress counseling, were more effective for the prevention of MI-induced PTSS/PTSD than current standard care of patients with high distress during MI. The need to find an effective treatment is essential as MI-induced PTSS might become chronic affecting the long-term prognosis of patients (9, 42). So far, multiple-session trauma-focused cognitive behavioral therapy provided within a few months after a trauma is recommended to reduce traumatic stress symptoms in patients with acute stress disorder or PTSD including MI-induced PTSD (43, 44). However, this type of intervention is cost-intensive and often not available. An early and individual single-session psychological counseling would therefore be preferable. Hence, future studies are needed to see if single-session counseling such as used in this trial, would yield such low PTSD prevalence rates.

Clinical and blinded assessment of MI-induced PTSS by both, clinician-rated PTSS and valid self-rated questionnaires, as well as the RCT design are strengths of this study. The longitudinal design with a follow-up period at 12 months is a further strength.

However, some limitations of our study should be mentioned as well. First, this was a single-center study with all of the inherent limitations of such a study design. Hence, generalization of findings to other hospitals and healthcare settings is limited. Second, due to the particular study design, i.e., performing counseling sessions at the emergency department, the counseling protocol had to be interrupted or even part of it postponed when the patient required other urgent examinations or medically indicated interventions, such as a coronary angiography. However, the idea of this approach was to provide a single counseling session explicitly for acute emergency cardiac situations. Finally, patients with severe somatic comorbidities and clinical depression who may be more vulnerable to develop PTSS had to be excluded.

To sum up, the findings of our study contribute to a better understanding of the effect of single-session counseling on clinician-rated MI-induced PTSD/PTSS. Particularly, we found that early trauma-focused counseling did not show any significant difference in clinician-rated PTSD/PTSS at 12 months follow-up when compared to an active control intervention in patients at risk to develop MI-induced PTSD. Interestingly, when comparing to previous studies, PTSS severity and prevalence of full PTSD prevalence were notably lower in the present study, suggesting a potential beneficial effect of both interventions. Further studies are needed to determine the most accurate approach of counseling.

## Data Availability Statement

The raw data supporting the conclusions of this article will be made available by the authors, without undue reservation.

## Ethics Statement

The studies involving human participants were reviewed and approved by Kantonale Ethikkommision Bern, Murtenstrasse 31, 3000 Bern. The patients/participants provided their written informed consent to participate in this study.

## Author Contributions

RK, J-PS, US, JB, and HZ contributed to the development of the study design. US, HZ, JB, RK, RL-M, and MP contributed to the development of the verum and control intervention. AP contributed to the discussion of the manuscript. KL and MP wrote the first draft of the manuscript. All authors critically revised and approved the final manuscript.

## Funding

The MI-SPRINT study is funded by the Swiss National Science Foundation (Project Number 140960, principal investigator: RK). Additional financial support comes from the Teaching and Research Directorate, Bern University Hospital, Switzerland. The funding bodies had no influence on the study design, in the writing of the manuscript, and in the decision to submit the manuscript for publication.

## Conflict of Interest

The authors declare that the research was conducted in the absence of any commercial or financial relationships that could be construed as a potential conflict of interest.

## Publisher's Note

All claims expressed in this article are solely those of the authors and do not necessarily represent those of their affiliated organizations, or those of the publisher, the editors and the reviewers. Any product that may be evaluated in this article, or claim that may be made by its manufacturer, is not guaranteed or endorsed by the publisher.
